# CCR5 Conformations Are Dynamic and Modulated by Localization, Trafficking and G Protein Association

**DOI:** 10.1371/journal.pone.0089056

**Published:** 2014-02-28

**Authors:** Ayanna J. Flegler, Gianguido C. Cianci, Thomas J. Hope

**Affiliations:** Department of Cell and Molecular Biology, Feinberg School of Medicine, Northwestern University, Chicago, Illinois, United States of America; University of Missouri, United States of America

## Abstract

CCR5 acts as the principal coreceptor during HIV-1 transmission and early stages of infection. Efficient HIV-1 entry requires a series of processes, many dependent on the conformational state of both viral envelope protein and cellular receptor. Monoclonal antibodies (MAbs) are able to identify different CCR5 conformations, allowing for their use as probes to distinguish CCR5 populations. Not all CCR5 MAbs are able to reduce HIV-1 infection, suggesting the use of select CCR5 populations for entry. In the U87.CD4.CCR5-GFP cell line, we used such HIV-1-restricting MAbs to probe the relation between localization, trafficking and G protein association for individual CCR5 conformations. We find that CCR5 conformations not only exhibit different localization and abundance patterns throughout the cell, but that they also display distinct sensitivities to endocytosis inhibition. Using chemokine analogs that vary in their HIV-1 inhibitory mechanisms, we also illustrate that responses to ligand engagement are conformation-specific. Additionally, we provide supporting evidence for the select sensitivity of conformations to G protein association. Characterizing the link between the function and dynamics of CCR5 populations has implications for understanding their selective targeting by HIV-1 and for the development of inhibitors that will block CCR5 utilization by the virus.

## Introduction

C-Chemokine Receptor 5 (CCR5) is a G protein-coupled receptor (GPCR) that also functions as the primary HIV-1 coreceptor during transmission and early stages of infection. The significance of CCR5 is highlighted by the fact that persons homozygous for a 32-base pair deletion (Δ32) within CCR5 harbor a truncated receptor that leads to resistance to HIV-1 acquisition [1], [2]. In its normal role, CCR5 functions in immune cell regulation by mediating lymphocyte trafficking to sites of inflammation [3], [4]. The immune function of CCR5 and increased susceptibility of Δ32 persons to West Nile virus highlights the importance of CCR5, suggesting that it is not completely dispensable without effect [5], [6]. Consequently, much emphasis has been placed on inhibiting HIV-1 utilization of surface CCR5 through the development of several CCR5 antagonists [7]–[9]. One such antagonist is Maraviroc (MVC), a CCR5 inhibitor approved for the treatment of HIV-1 infection, which blocks receptor activation without masking the binding sites for chemokines and the HIV envelope glycoprotein [7], [10], [11]. In contrast, CCR5 chemokines Macrophage Inflammatory Protein-1α (MIP-1α), MIP-1β, Monocyte chemotactic protein (MCP)-2 and RANTES naturally restrict HIV-1 infection by masking virus binding sites and promoting CCR5 cell surface down-modulation [12], [13]. Several groups have exploited the natural chemokine mechanisms of restriction by modifying the N-terminus of RANTES to produce CCR5 small-molecule inhibitors. Such RANTES analogs have different effects on CCR5 internalization and signaling, and have also been demonstrated to be potent HIV-1 inhibitors [14]–[18].

CCR5 activation occurs upon ligand binding and subsequent exchange of GTP for GDP in the receptor-bound Gα subunit of the G_i_α class of heterotrimeric G proteins, a pertussis toxin-sensitive process [19]. Receptor activation precedes a multi-step desensitization process leading to β-arrestin binding and to receptor uncoupling from the G protein [20], [21]. Phosphorylated serine residues on the CCR5 cytoplasmic tail serve as a binding site for β-arrestin, an adaptor protein involved in the translocation of receptors from the cell surface to internal compartments via clathrin-meditated endocytosis [22], [23]. The route of CCR5 trafficking has been well characterized. After chemokine engagement, the receptor is internalized and travels to the endosomal recycling compartment (ERC), from the ERC to the trans Golgi network (TGN), and then back to the plasma membrane [24]–[26].

Ligand binding to GPCRs can achieve varying efficacy for downstream signaling depending on the conformation state of the receptor [27], [28]. The existence of distinct CCR5 conformations is seen through discordant binding levels of ligands and antibody affinities of CCR5 specific monoclonal antibodies (MAbs) [29]–[31]. A recent report has characterized CCR5 receptor populations by illustrating their differential engagement based on having high or low affinity for chemokines [32]. On the one hand, competitive CCR5 binding experiments revealed that native chemokines bound with low affinity to gp120-binding receptors, an explanation for why such chemokines exhibit weak inhibition of HIV. On the other hand, chemokine analogs bound with high affinity to the same receptors [32]. Additionally, coupling assays revealed that gp120 binds indiscriminately to ^NF^G protein-coupled and -uncoupled receptors [32]. Other data highlights the selective usage of CCR5 conformations by HIV with the observation that only a subset of MAbs restricts infection [31].

Much is known about CCR5 trafficking and the identity of CCR5 conformational heterogeneity, but it is not clear if the normal cycling of the receptor influences CCR5 conformations. Additionally, it is not known if the CCR5 conformation state is static or readily altered. Former studies have demonstrated the HIV-1-restricting and gp120-antagonizing effect of MAbs 2D7, 45531, PA11 and MC-5 [29]–[31]. Using these MAbs as probes to detect CCR5 conformations, we investigated whether a distinction exists between conformations and their localization, trafficking, ligand engagement and G protein association. We observe that CCR5 populations vary in their abundance and spatial distribution throughout the cell when aspects of normal function are modulated. We find that conformations display distinct sensitivities to endocytosis inhibition. Furthermore, RANTES analogs differentially alter surface and internal levels of CCR5 conformations. Distinguishing CCR5 conformational heterogeneity relative to receptor functions has implications for understanding their selective use by HIV-1 and the development of improved strategies to block CCR5 use by HIV-1.

## Materials and Methods

### Antibodies

CCR5 MAbs 2D7 and 45531 were purchased from R&D Systems (Minneapolis, MN, USA) and BD Pharmingen (San Jose, California, USA) respectively. MC-5 monoclonal CCR5 antibody was a gift from Matthias Mack at the University of Regensburg in Germany. Separate stocks of PA11 were received from John Moore at Cornell University. TRITC secondary antibodies were purchased from Jackson ImmunoResearch (West Grove, PA, USA).

### Cells and reagents

The following reagent was obtained through the NIH AIDS Reagent Program, Division of AIDS, NIAID, NIH: U87.CD4 from Dr. HongKui Deng and Dr. Dan R. Littman. CCR5-GFP was stably expressed in U87.CD4 cells using a lentivirus-based strategy. Briefly, packaging plasmid ΔNRF, retroviral vector pBABE.CCR5-GFP and VSV-G were cotransfected into 293T cells. After 48 hours, supernatants were harvested and used to transduce U87.CD4 cells. The polyclonal population was sorted by FACS for a low-expressing GFP cell population. The CCR5-GFP fusion product was found to remain intact after protein synthesis (Figure S1). U87.CD4.CCR5-GFP cells were maintained in DMEM (Cellgro, Manassass, VA USA) with 10% FBS and 2µg/mL puromycin (InvivoGen, San Diego, California, USA). The puromycin concentration was based on previous cytotoxicity assays. 293-Affinofile cells were maintained in DMEM supplemented with 10% FBS and 50µg/mL Blasticidin S HCl (Invitrogen, Grand Island, NY). To induce CCR5 expression, 293-Affinofile cells were treated with either 0.25µM or 1.0µM Ponasterone (Invitrogen) for 24 hours prior to experimental setup. Chlorpromazine (Sigma, St. Louis, MO, USA) and sucrose (VWR, Radnor, PA, USA) were dissolved in distilled water. Dynasore (Enzo Life Sciences, Farmingdale, NY, USA) and Nystatin (Sigma) were dissolved in DMSO.

### Immunofluorescence staining

Cells were plated on 12 mm glass cover slips and allowed to adhere overnight. *Surface staining*- Live cells were temperature-shifted from 37°C to 4°C to reduce lipid bilayer mobility and prevent receptor internalization. Cells were then treated with block containing 5% Normal Donkey Serum in 1X PBS for 20 min at 4°C to block non-specific Ab binding sites. Cells were surface stained with unconjugated CCR5 MAbs for 1 hr at 4°C, then washed. Cells were subsequently incubated with a TRITC-labeled secondary antibody at 4°C for 30 min at a concentration yielding maximal fluorescence for all MAbs and background fluorescence in the absence of MAb. MAb concentrations were titrated in U87.CD4 cells lacking CCR5 and chosen based on the highest concentration that exhibited a fluorescent intensity comparable to background. After antibody staining, cells were incubated with Hoechst for visualization of nuclei. *Total staining*- Cells were stained similarly to the above protocol, with the addition of Triton X-100 at 0.1% in the blocking buffer. Staining was also performed at room temperature.

### Image capture

Images were collected in a z-series, 0.2µM apart, on a DeltaVision (Applied Precision) microscope. Following acquisition, images were deconvolved using softWoRx (Applied Precision) deconvolution software. For each condition, at least 30 images were captured at 100× magnification.

### Image quantification

To quantify overlap of fluorophores, an Interactive Data Language (IDL) program was developed and used for all images. The CCR5-GFP threshold was set above the non-specific signal from GFP-negative cells to neglect background voxels (volumetric pixels) and emphasize internal regions and membrane structures. The GFP-positive voxels were analyzed for overlap with TRITC-labeled secondary antibodies. For each condition, one image was chosen to establish TRITC brightness thresholds. The threshold in the TRITC channel was set above secondary-only background. After establishing thresholds, the IDL program calculated the percentage overlap of the voxels above both thresholds on a per image basis. The program applies the same threshold values to all images from the same condition thus avoiding user bias and providing quick analysis. The graphed values are the average percent overlap of all cells for a given condition across experiments.

### Statistical analysis

Statistical analysis was performed on GraphPad Prism 5 (GraphPad Software, La Jolla, CA). Graphed values are averages with error bars depicting ± SEM.

## Results

### CCR5 conformations are distributed differentially within a CCR5-GFP expressing cell line

**Figure 1:**
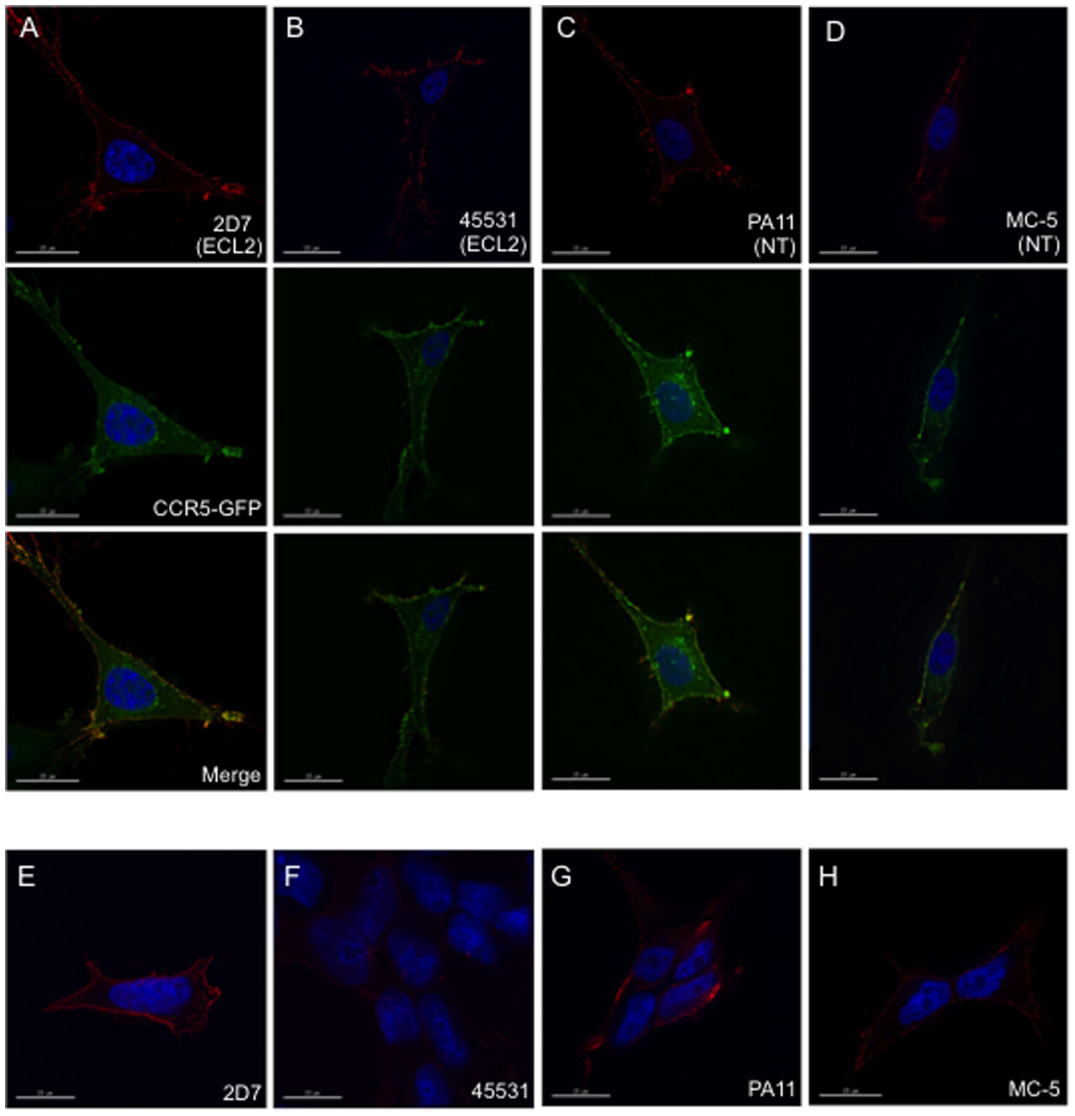
Surface CCR5 conformation localization within U87.CD4.CCR5-GFP cells. Representative images from (A-D) U87.CD4.CCR5-GFP or (E-H) 293-Affinofile cells stained with the indicated CCR5 MAbs (red). CCR5 MAb 45531 exhibited a more punctate staining pattern than other MAbs. Total is expressed by CCR5-GFP (green) and is demonstrated by fluorescence on surface and internal cellular compartments. Images are displayed as middle Z sections.

The presence and abundance of CCR5 in the cell membrane are crucial for HIV binding and entry. Using a panel of CCR5 MAbs that were inhibitory to various HIV-1 isolates as probes for CCR5 conformations [31], we investigated whether the cellular distribution of such surface CCR5 populations was distinct. To assess the abundance of various conformations relative to total CCR5, we generated the U87.CD4.CCR5-GFP stable cell line that expresses GFP on the C-terminal end of CCR5, thereby preventing interference with ligand binding. MAbs 45531 and 2D7 bind the Extracellular Loop 2 (ECL2) of CCR5 while MC-5 and PA11 both bind the N-terminal (NT) epitope, with PA11 recognizing a Tyr-sulfated CCR5 NT. Surface staining in U87.CD4.CCR5-GFP cells with these MAbs generated two distinct distribution patterns. MAb 45531 was previously demonstrated to stain in clusters and bind to membrane regions high in cholesterol [31].We similarly observed that MAb 45531, as well as MC-5, typically bound in select regions of the plasma membrane while all other MAbs were more abundant and uniformly distributed throughout the surface of U87.CD4.CCR5-GFP cells (Figure 1A-D). To determine whether the observed staining patterns were unique to the U87 cell line, we also performed surface staining in 293-Affinofile cells, a dually CD4- and CCR5-inducible HEK 293 cell line [33]. 293-Affinofile cell surface staining with CCR5 MAbs following treatment with 0.25µM Ponasterone A to induce low CCR5 expression results in a similar staining pattern as seen in U87.CD4.CCR5-GFP cells (Figure 1E-H). Specifically, the uniform staining pattern seen with MAbs 2D7 and PA11 was similar to that in U87 cells. This was distinct from MAbs MC-5 and 45531, as both appeared less intense in fluorescence, while 45531 staining also appeared more punctate (Figure 1F). Using a saturating concentration of Ponasterone A, we observed an expected increase in CCR5 protein expression to correspond with elevated levels of surface CCR5 conformations as detected by MAbs (Figure S2).

**Figure 2:**
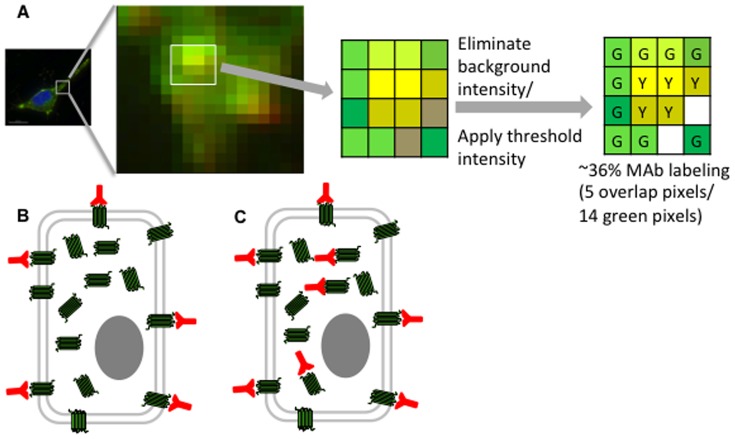
Method of quantification for CCR5-GFP cells. (A) Pixels from single cell images are both chosen or eliminated based on a selected fluorescent intensity. The resulting ‘positive’ pixels in each wavelength are used to calculate the percentage of MAb labeling. Percent (%) CCR5 positive for MAb labeling  =  pixels positive for MAb (red) and CCR5 (green)/pixels positive for CCR5 (green). (B) Example MAb labeling at the cell surface (surface labeling of total CCR5 molecules  = 30%). (C) Example MAb total labeling (total labeling of total CCR5 molecules  = 53%).

**Figure 3:**
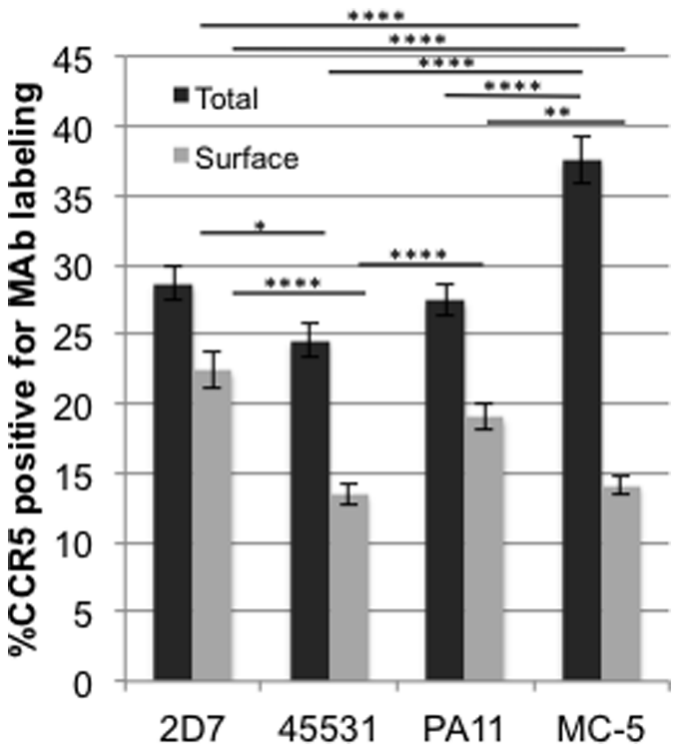
Surface and Total CCR5 conformation expression within U87.CD4.CCR5-GFP cells. U87.CD4.CCR5-GFP cells were incubated with 2D7, 45531, PA11 or MC-5 for surface (grey bars) or total labeling (black bars). CCR5 MAb labeling was measured using an IDL program and represented as a percentage of total CCR5. Graphs display averages of 4 independent experiments (N = 30 cells per experimental condition). Statistical analysis was performed using an unpaired *t* test. Error bars represent Standard Error of the Mean (SEM), ^****^p<.0001, ^**^p<.01, ^*^p<.05

To quantify MAb labeling of the CCR5-GFP fusion protein, we measured the voxel-based (x,y,z) overlap of TRITC and GFP based on brightness thresholds using an Interactive Data Language (IDL) program (Figure 2). We found that the quantified data complements our visual observations of surface MAb staining. For example, the limited cell surface labeling seen with MAb 45531 was reflected in our quantitative analysis by its percentage labeling total GFP being the least among all MAbs (Figure 3). Additionally, the abundance of surface conformations detected by PA11 and 2D7 were significantly different from MAb 45531 and MC-5 (Figure 3). We also performed staining after cell permeabilization to assess the total presence of each conformation on the surface and within the cell relative to the total CCR5 population. Total staining also allows us to determine the abundance of conformations existing internally by extracting the difference between total and surface labeling percentages for a given MAb (Figure 2B and C). Conformations detected by MAbs 2D7, 45531 and PA11 were highly distributed on the cell surface, accounting for 78, 54 and 69% of each total subpopulation, respectively. The CCR5 population labeled with MC-5 however, had less than half of its expression at the cell surface (14% of 37% total CCR5), indicating that the majority of the conformation existed internally (Figure 3). Taken together, our results highlight the ability of CCR5 conformations to be differentially distinguished in the cell utilizing our antibody panel.

### Endocytosis inhibition has varying effects on CCR5 conformations

**Figure 4:**
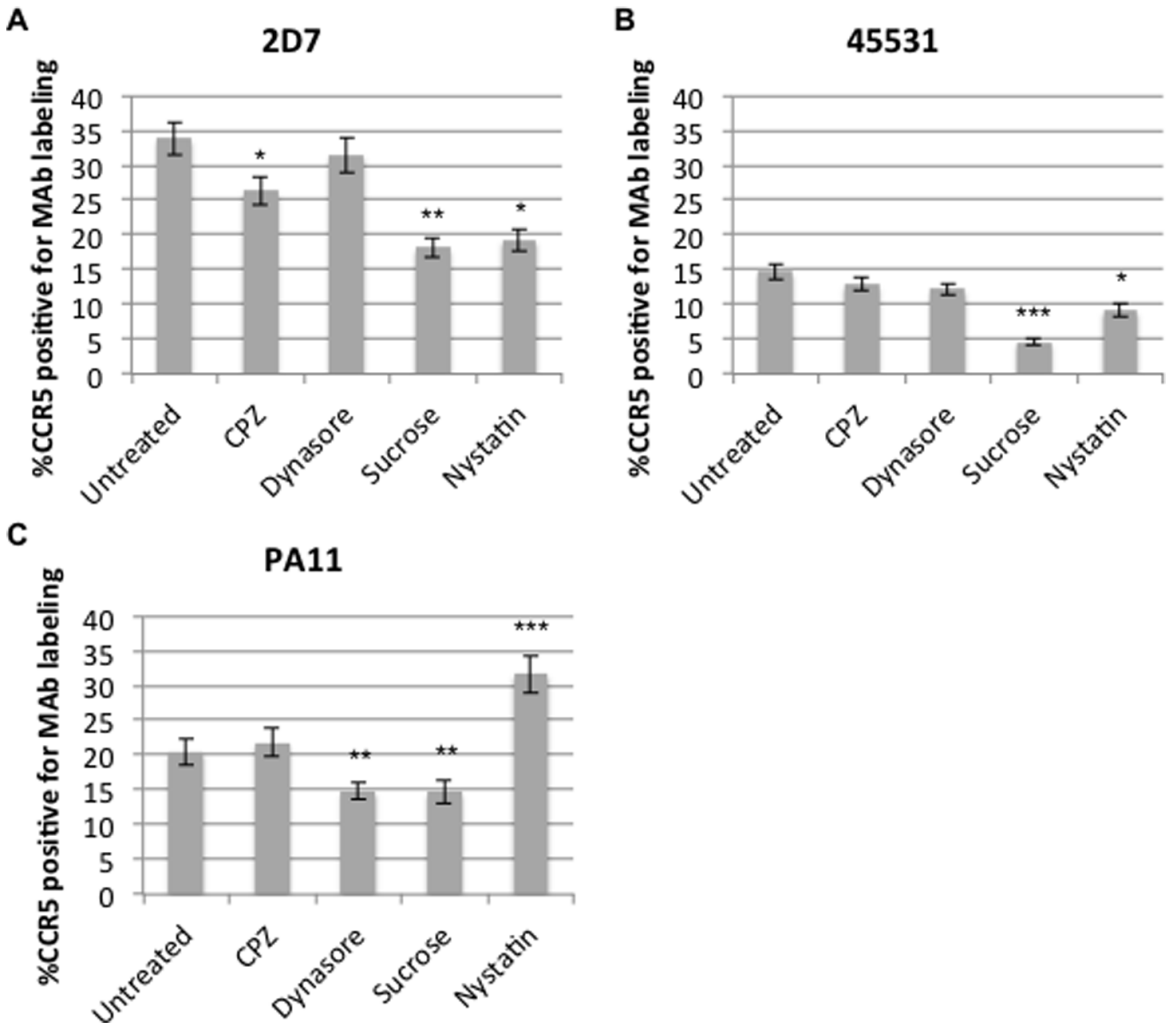
Surface expression of CCR5 conformations upon blocking endocytosis. U87.CD4.CCR5-GFP cells were treated with 0.45 M sucrose, 5µM Dynasore, 5µg/mL chlorpromazine, or 50µg/mL nystatin, inhibitors of both clathrin- and caveolae-mediated endocytosis. Cells were then surface stained with (A) 2D7, (B) 45531 or (C) PA11. Graphs display averages of 3 experiments (N = 30 per experimental condition). Statistical analysis was performed using analysis of variance (ANOVA). ^***^p<.001, ^**^p<.01, ^*^p<.05, where significantly different than ‘Untreated.’

In preliminary experiments aimed to visualize the localization of CCR5 conformations, only certain MAbs demonstrated punctate staining near microvilli structures, an area of dense cross-linked actin filaments which contribute to processes such as endocytosis [34]. We were therefore interested in whether CCR5 conformations differed in their early trafficking, particularly at endocytosis, and examined this possibility by measuring changes in MAb labeling after disrupting endocytotic pathways. To block endocytosis we used sucrose, chlorpromazine and dynasore. Sucrose depletes cytoplasmic pools of clathrin by causing an accumulation of clathrin microcages on the inner membrane of cells and disrupting the normal lattice formation [35]. Chlorpromazine (CPZ) prevents recycling of clathrin by sequestering it near endosomal membranes, whereas dynasore inhibits cellular use of select dynamins used for clathrin- and caveolae-mediated endocytosis pathways [36]–[38]. Cells were treated with an endocytosis inhibitor for 1 hour at 37°C then surface stained as described in Materials and Methods. Reagent concentrations were chosen based on their ability to restrict transferrin uptake into cells (Figure S3). A significant reduction in surface labeling occurred for all MAbs upon sucrose treatment, but only for MAbs 2D7 and PA11 after CPZ and dynasore incubation, respectively (Figure 4). We also investigated whether conformations would be sensitive to nystatin, an inhibitor of caveolae-mediated endocytosis, as some reports show a role of caveolae in CCR5 trafficking [39], [40]. Exposure to nystatin led to a reduction in conformations detected by ECL2-binding MAbs 2D7 and 45531and an increase in PA11 staining (Figure 4A, B). However, these alterations seemed not to be a consequence of caveolae sequestration as dynasore, also an inhibitor of caveolae-mediated endocytosis, did not alter binding (Figure 4). Instead, nystatin-mediated changes in CCR5 conformations are likely due to its effect on membrane cholesterol levels, which has been linked to CCR5 localization and mobility [31], [41]. These data show that levels of CCR5 conformations used by HIV-1 are differentially altered upon interrupting cellular trafficking, specifically the internalization of molecules.

### Engagement of CCR5 conformations by HIV-1-inhibitory peptides leads to conformational changes

The dynamic nature of CCR5 has been suggested through changes in receptor conformation upon MVC binding to transmembrane regions [10]. However, how MVC and HIV inhibitors that bind to extracellular CCR5 domains impact the presence of existing conformations remains poorly defined. To probe the effects of ligand engagement on CCR5 conformations, we used two RANTES analogs, 5P12 and 5P14, which restrict HIV-1 infection (Figure S4A and [15]). Analogs generated from N-terminal modifications of RANTES yield varying degrees of CCR5 binding, signaling and internalization [14], [15]. Although neither 5P12 nor 5P14 generate GPCR signaling activity upon CCR5 binding, 5P14 has been demonstrated to induce CCR5 internalization (Figure S4B and [15]). An essential binding site for CCR5 ligands and RANTES analogs resides in the ECL2 of CCR5 [42]. Since the ECL2 is also the epitope of MAbs 45531 and 2D7, epitope masking occurs when analogs are allowed to first bind CCR5 (Figure S4C). We therefore focused our engagement studies on conformations detected by MAbs PA11 and MC-5, as their NT binding can still be detected following analog treatment (Figure S4C).

**Figure 5:**
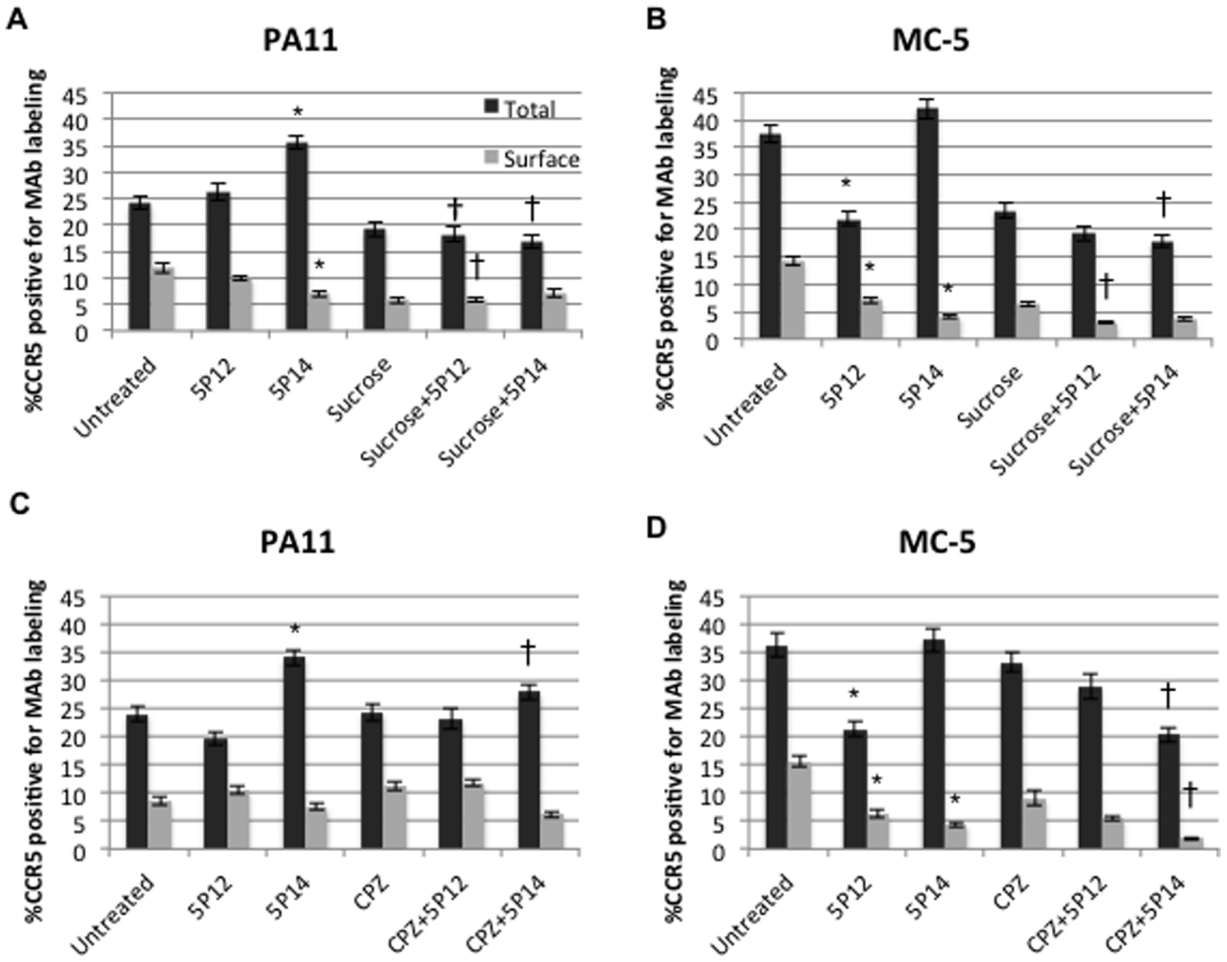
CCR5 conformation changes upon engagement. U87.CD4.CCR5-GFP cells were treated with 100 nM RANTES analogs, 0.45 M sucrose or 5µg/mL chlorpromazine, or pretreated with an endocytosis inhibitor followed by addition of analog in inhibitor-containing medium. Cells were then stained for surface (grey bars) or total (black bars) conformation expression using MAb PA11 (A,C) or MC-5 (B,D). Graphs display averages of 2 (B,D) or 4 (A,C) experiments (N = 35 per experimental condition). Statistical analysis was performed using analysis of variance (ANOVA). ^*^p<.05 between untreated and analog-treated, ^†^p<.05 between analog-treated only and in combination with endocytosis inhibitor.

Treatment with 5P14 leads to reduced levels of surface conformations detected by MC-5 and PA11, an observation consistent with the internalization property of this analog [15]. Although 5P14 induces CCR5 sequestration, the level of internalization is not as high as other analogs, such as PSC-RANTES [15]. This was demonstrated by the partial reduction of surface conformations after 5P14 treatment (Figure 5, grey bars and Figure S4B). We expected the observed 5P14-induced reduction of surface conformations to not affect total levels of MAb labeling, but to instead generate an increase in internal conformations due to internalization. Indeed, with MAb MC-5 the percentages of internal conformation detected increased from 23% to 38% (Figure 5B) and from 20% to 32% (Figure 5D). Notably, 5P14-induced internalization led to increased levels of total PA11 MAb detection (Figure 5), implying an exposure of additional internal conformations either during the path to becoming internalized or once within internal cellular compartments. Surprisingly, treatment with non-CCR5-internalizing 5P12 also reduced the surface and total conformation detected by MC-5 (Figure 5B and D), but not with PA11. The decrease in total MC-5 labeling after 5P12 treatment is likely a result of reduction in the surface conformation detected by this antibody. The unexpected surface reduction of only MC-5 by 5P12 binding illustrates that changes of select conformations upon engagement is both dynamic and distinct.

Several studies implicate internalization via clathrin-coated pits after CCR5 agonist binding [39], [43]. We further investigated whether the 5P12 and 5P14 engaging mechanism is sensitive to clathrin-mediated endocytosis, and whether this sensitivity is distinct for specific CCR5 conformations. Cells were either treated with analogs or endocytosis inhibitors alone, or pretreated with endocytosis inhibitors followed by addition of analogs, and then stained for surface and total conformations as described in Materials and Methods. For surface and total staining, we observed reduced labeling of both MC-5 and PA11 when treated with sucrose, and unchanged labeling upon CPZ treatment, both observations consistent with previous experiments (Figure 4). We focused on comparing conditions with analog alone and analog after pretreatment with endocytosis inhibitors to gauge the effect of internalization after analog engagement. Sucrose significantly reduced both PA11 and MC-5 5P12-engaged surface labeling, whereas there was no change when combined with 5P14 (Figure 5 A and B; grey bars). This suggests that the 5P14-mediated internalization of the probed surface conformations is not likely altered by inhibiting clathrin-mediated endocytosis. The loss of surface conformations upon combined sucrose and 5P12 treatment is likely due to sucrose, an effect seen when comparing untreated with sucrose-treated cells (Figure 5), and in former experiments using other MAbs (Figure 4). This trend demonstrates the consistent sensitivity of various CCR5 conformations to modifications of the endocytotic machinery. For both MAbs, pretreatment with CPZ did not considerably alter surface labeling upon 5P12 and 5P14 engagement (Figure 5 C and D; grey bars). However, a significant reduction in total labeling for both MAbs occurred when combining inhibitors with 5P14 (Figure 5; black bars) to a much greater extent than the reduction at the cell surface, demonstrating an overall decrease in internal levels. Such results have implications for trafficking on CCR5 internal conformations when they are engaged with ligands.

### G protein uncoupling from CCR5 can alter surface CCR5 conformations

**Figure 6:**
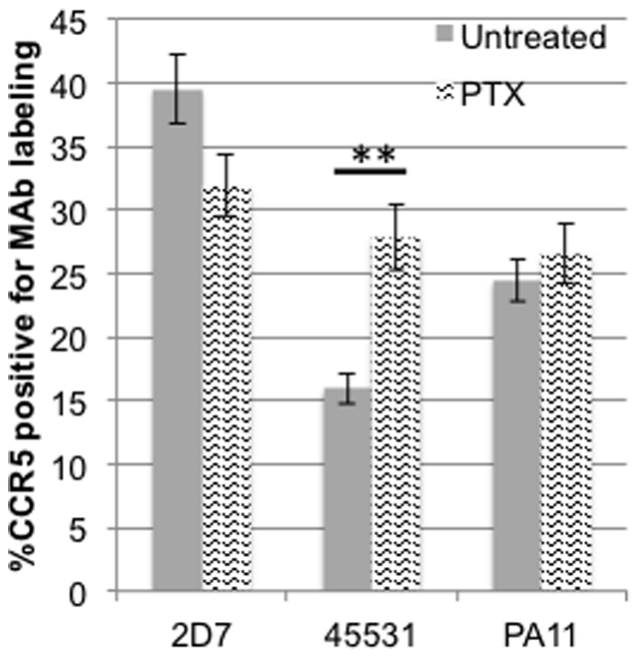
Surface CCR5 conformation is sensitive to pertussis toxin treatment. Cells were treated with 1µg/mL pertussis toxin and then surface stained with MAbs 2D7, 45531 or PA11. Changes in MAb 45531 labeling represent sensitivity to PTX treatment. Statistical analysis was performed using a paired student's t-test. ^**^p<.01

CCR5 has been shown to become stabilized in a high-affinity conformation for agonists upon coupling to ^NF^G proteins [32]. Our observation that ligands can affect the surface exposure of CCR5 conformations led us to investigate whether such external responses can be manipulated through intracellular changes within CCR5 domains, specifically their association with G proteins. We treated U87.CD4.CCR5-GFP cells with 1µg/mL pertussis toxin (PTX) for 1 hour at 37°C to prevent the activation of the G_αi_ protein, and then surface stained using CCR5 MAbs. PTX treatment increased the labeling with MAb 45531, but not with MAbs 2D7 and PA11 (Figure 6). This result was particularly surprising due to the lack of agonist engagement, which would lead to PTX-sensitive G protein coupling and subsequent receptor activation. Instead, our data illustrates that disruption of the G protein receptor association, even for unbound CCR5 conformations, can affect receptor population levels at the cell surface.

## Discussion

Understanding how trafficking and signaling influences CCR5 populations is important for understanding CCR5 biology and HIV-1 pathogenesis. With knowledge that CCR5 antibodies can bind distinct CCR5 conformational populations, our research used MAbs as probes to investigate the spatial distribution and changes among CCR5 populations upon altering CCR5 trafficking and receptor association with G_i_α. To this end, we established a quantification method that utilized individual voxel fluorescence intensities to measure MAb staining relative to total CCR5 present within a cell. Visual and quantitative analyses demonstrate the existence of CCR5 conformations through disparate surface and total labeling among CCR5 MAbs. MAb 45531 detected a conformation whose surface expression was reduced compared to other highly expressed CCR5 conformations. Inhibition studies demonstrated that MAb 45531 restricts Vicriviroc (VVC)-resistant HIV-1 and not the VVC-sensitive parental HIV-1 strain, whereas MAbs 2D7 and PA11 were able to restrict parental and to some extent VVC-resistant strains [31]. These data support the view that certain CCR5 conformations are used selectively and suggest that HIV-1 utilizes conformations most abundant on the cell surface, a possible means to increase the likelihood of coreceptor binding and entry. However, in the case of selective pressure, as with CCR5 inhibitor-resistant viruses, HIV-1 is capable of utilizing CCR5 conformations having limited presence on the cell surface, an adaptation likely to maximize chances of binding.

Using RANTES analogs possessing antiviral potency, we investigated whether their engagement promotes different responses between CCR5 conformations. Binding of PSC-RANTES to CCR5 creates a durable association that leads to long-term sequestration within internalized compartments, a result potentially due to exposing a particular CCR5 conformation that is unfavorable for trafficking back to the cell surface [25]. Coinciding with this, our results show that 5P14 also appears to play a role in altering internal conformations. While characterization of 5P14 has determined that CCR5 down-modulation is reason for its inhibitory mechanism [15], identifying whether its ability to internally expose new conformations is conserved during routes back to the plasma membrane can be a topic for future investigations. Understanding the relationship between the ability of analogs to restrict infection and their effect on CCR5 conformations is important for the optimization of more effective inhibitory agents. Ideally, it would be beneficial to develop an analog that promotes CCR5 internalization, but not agonist activity, and is able to restore to the cell surface CCR5 conformations not highly utilized by HIV-1. Analog 5P12 has been characterized as a potent anti-HIV molecule, yet the restriction mechanism has not been fully defined [15]. In our studies, we observed differences in the response of MC-5- and PA11-detecting conformations to 5P12, a potential insight into the restrictive activity of 5P12. This result may also have been due to PA11 detecting a Tyr-sulfated NT [31], as CCR5 post-translational modifications of extracellular domains have been demonstrated to influence chemokine binding [44]. As the binding of chemokine analogs to high-affinity CCR5 populations has been recently demonstrated [32], our studies additionally highlight the CCR5 conformation heterogeneity in responses to ligand engagement.

Investigating constitutive and agonist-induced internalization has implications for understanding trafficking mechanisms that will eventually lead to resurfacing of CCR5 to the plasma membrane, an important topic as the presence of select CCR5 populations is beneficial for HIV-1 binding. Cellular treatments with endocytosis inhibitors led to changes within surface conformations, and more notably with the internal PA11-detecting conformation upon 5P14 treatment in comparison to analog treatment alone (Figures 4 and 5). The sensitivities to inhibitors were most significant and consistent with sucrose, as collectively, conformations were not significantly altered when clathrin-mediated endocytosis was inhibited. The sucrose endocytosis inhibition mechanism may therefore provide insight into the use of specific adaptor molecules by CCR5 conformations for trafficking, an area for future investigations. Our results did not implicate use of caveolae-mediated endocytosis for constitutive internalization of the probed CCR5 conformations. Loss of CCR5 internalization has been illustrated with filipin, an inhibitor of caveolae-mediated endocytosis that, like nystatin, also sequesters membrane cholesterol [40]. The association of cholesterol with CCR5 has been demonstrated with evidence showing its necessity for CCR5 mobility [41], and with recent evidence suggesting the presence of a CCR5 conformation in cholesterol-rich lipid-rafts [31]. The observed increase in PA11 MAb labeling upon nystatin treatment is also likely due to the cholesterol sequestering effect of this compound, and provides support for select CCR5 conformations existing in membrane regions enriched in cholesterol.

Studies demonstrate that GPCRs and G proteins can exist in a precoupled state [45]–[47], and that interaction of G proteins with CCR5 can influence ligand and HIV-1 binding [48], [49]. In addition to the unique labeling and HIV-1 restriction of MAb 45531, PTX treatment only affected MAb 45531 labeling (Figure 6). Other data demonstrate that cholesterol in membranes is essential for CCR5 signaling [40]. As the conformation detected by MAb 45531 may already exist in cholesterol-rich regions [31] and is likely most associated with G proteins, future studies can investigate its signaling in comparison to other conformations. Although recent data demonstrates that select CCR5 MAbs, one being 45531, can exhibit some nonspecific binding [50], our quantification method detects MAb positive voxels overlapping with CCR5/GFP, minimizing the inclusion of any off-target binding. Since the MAbs used in our research display some level of restriction to parental or VVC-inhibitor-resistant HIV-1 infection, our studies shed light on the use and characteristics of distinct CCR5 conformations with relevance to their utilization by HIV-1. Additionally, our results underscore the importance of the diversity of CCR5 antibodies and their ability to detect different CCR5 conformations. Clarity in understanding the use of CCR5 conformations can be increased in future studies investigating their presence in primary cells. Moreover, future studies investigating CCR5 should consider the existence of multiple conformations during analysis.

## Supporting Information

Figure S1 **CCR5-GFP protein is intact within U87.CD4.CCR5-GFP cells.** Lysates were collected from non-CCR5-expressing 293T and Affinofile cells as controls. Affinofile cells were treated 24 hours with 1µM Ponasterone to induce high expression of CCR5.
(TIFF)

Figure S2 **Increased CCR5 protein expression leads to elevated levels of surface CCR5 conformations.** Affinofile cells were treated with a saturating concentration of 1µM Ponasterone for 24 hours before surface staining with the indicated MAbs (red).
(TIFF)

Figure S3 **Inhibitors of clathrin-mediated endocytosis blocks transferrin uptake in U87.CD4.CCR5-GFP cells.** Cells were treated for 45 min at 37°C with inhibitors, then incubated with fluorescently labeled transferrin (red).
(TIFF)

Figure S4 **Effects of RANTES analog treatment.** U87.CD4.CCR5 cells were infected with HIV-GFP in the presence or absence of analog for 4 hours (A). Cells were incubated with RANTES analogs for one hour at 37°C prior to surface staining with the indicated MAbs. Analogs were found to vary in their ability to cause CCR5 internalization (B). Following addition of RANTES analogs, cells were infected with HIV-1. Infection was carried out for 48 hours. Cells were analyzed by flow cytometry to determine the GFP^+^ cell population. RANTES analogs mask binding of CCR5 MAbs specific for ECL2 and not NT epitope (C). Statistical analysis was performed using analysis of variance (ANOVA). ^***^p<.001
(TIFF)
