# Supplementary figures and images for: CCR5 Conformations Are Dynamic and Modulated by Localization, Trafficking and G Protein Association
Source: PLoS One. 2014 Feb 28;9(2):e89056. doi: 10.1371/journal.pone.0089056 (PMC3938464; doi:10.1371/journal.pone.0089056)

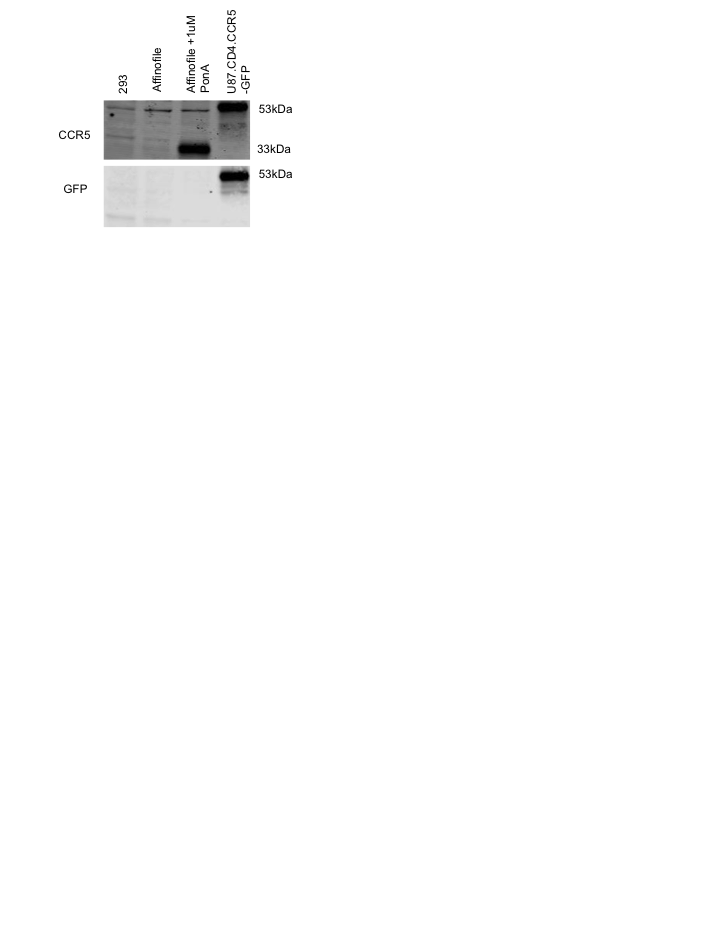

Supplement: Figure S1 — CCR5-GFP protein is intact within U87.CD4.CCR5-GFP cells. Lysates were collected from non-CCR5-expressing 293T and Affinofile cells as controls. Affinofile cells were treated 24 hours with 1µM Ponasterone to induce high expression of CCR5. (TIFF) [file pone.0089056.s001.tif]

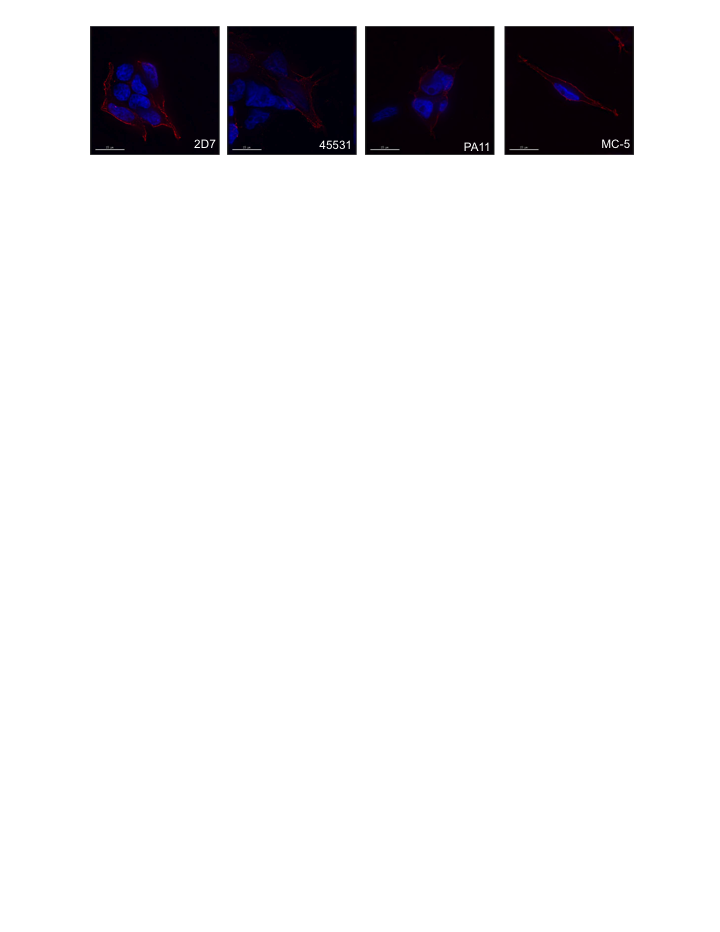

Supplement: Figure S2 — Increased CCR5 protein expression leads to elevated levels of surface CCR5 conformations. Affinofile cells were treated with a saturating concentration of 1µM Ponasterone for 24 hours before surface staining with the indicated MAbs (red). (TIFF) [file pone.0089056.s002.tif]

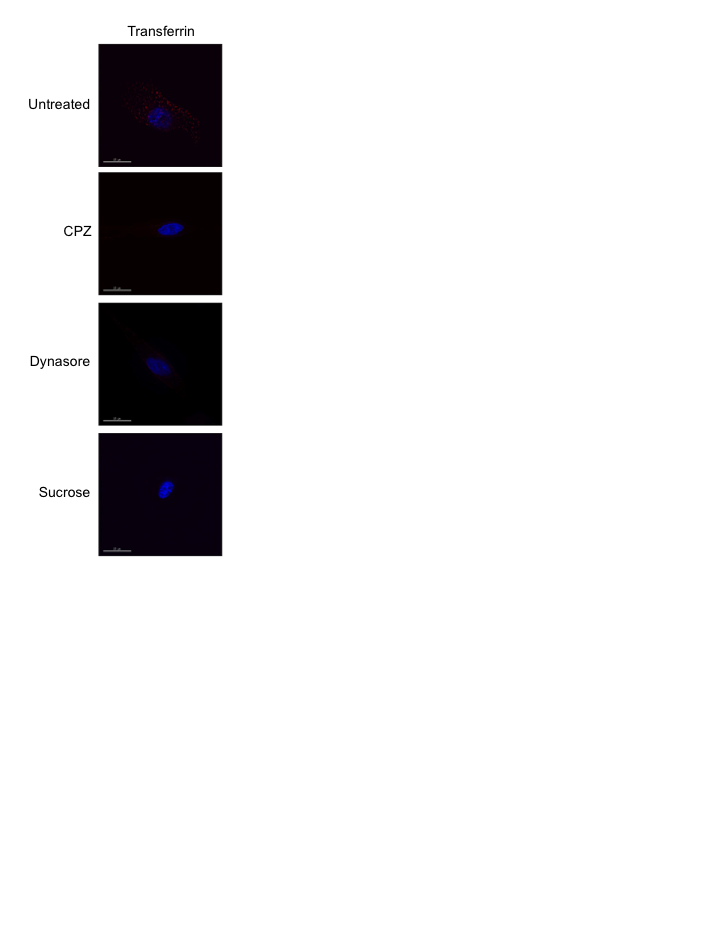

Supplement: Figure S3 — Inhibitors of clathrin-mediated endocytosis blocks transferrin uptake in U87.CD4.CCR5-GFP cells. Cells were treated for 45 min at 37°C with inhibitors, then incubated with fluorescently labeled transferrin (red). (TIFF) [file pone.0089056.s003.tif]

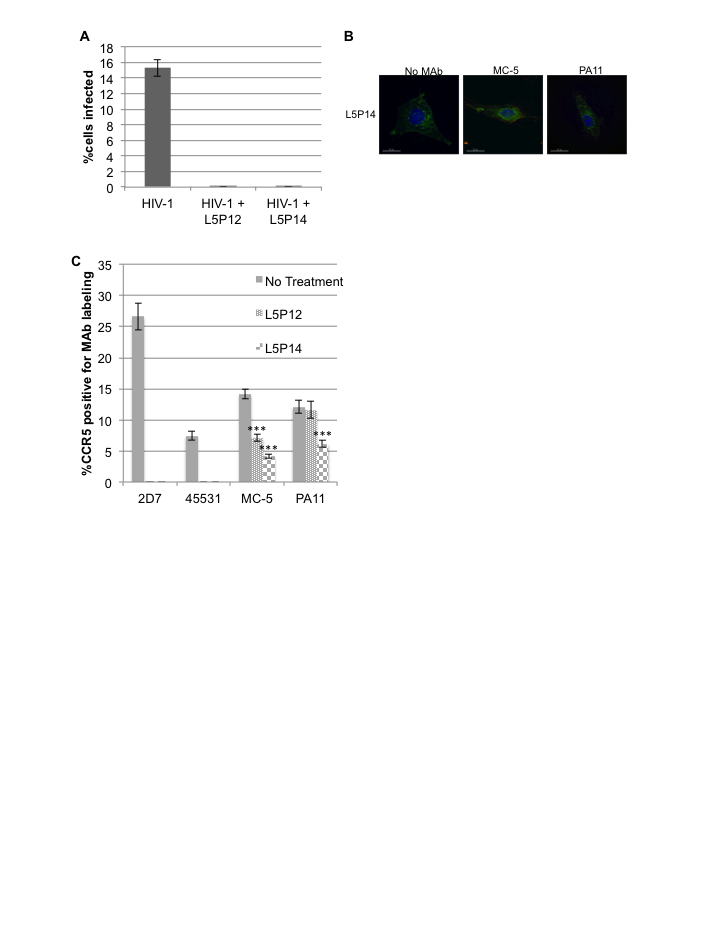

Supplement: Figure S4 — Effects of RANTES analog treatment. U87.CD4.CCR5 cells were infected with HIV-GFP in the presence or absence of analog for 4 hours (A). Cells were incubated with RANTES analogs for one hour at 37°C prior to surface staining with the indicated MAbs. Analogs were found to vary in their ability to cause CCR5 internalization (B). Following addition of RANTES analogs, cells were infected with HIV-1. Infection was carried out for 48 hours. Cells were analyzed by flow cytometry to determine the GFP+ cell population. RANTES analogs mask binding of CCR5 MAbs specific for ECL2 and not NT epitope (C). Statistical analysis was performed using analysis of variance (ANOVA). ***p<.001 (TIFF) [file pone.0089056.s004.tif]
